# A Better Understanding of Moyamoya in Trisomy 21: A Systematic Review

**DOI:** 10.7759/cureus.23502

**Published:** 2022-03-26

**Authors:** Arowa Abdelgadir, Hamna Akram, Maurice H Dick, Nabeel R Ahmed, Abanti Chatterjee, Sushil Pokhrel, Vaishnavi Vijaya Kulkarni, Safeera Khan

**Affiliations:** 1 Clinical Research, California Institute of Behavioral Neurosciences & Psychology, California, USA; 2 Clinical Research, California Institute of Behavioral Neurosciences & Psychology, Park Ridge, USA; 3 Clinical Research, California Institute of Behavioral Neurosciences & Psychology, Fairfield, USA; 4 Clinical Research, California Institute of Behavioral Neurosciences & Psychology, Kolkata, IND; 5 Clinical Research, California Institute of Behavioral Neurosciences & Psychology, Binghamton, USA; 6 Internal Medicine, California Institute of Behavioral Neurosciences & Psychology, Fairfield, USA

**Keywords:** down syndrome(ds), pathogenesis of moyamoya disease, moyamoya angiopathy, moyamoya disease (mmd), down's syndrome

## Abstract

Moyamoya disease is defined as stenosis of the internal carotid artery or the middle, anterior or posterior cerebral arteries with considerable collateral development. This collateral vessel has a particular appearance in angiographic examinations. Moyamoya syndrome is a term used to describe when moyamoya disease occurs in conjunction with other systemic disorders. One of the associations is Down syndrome. Moyamoya syndrome is very common in patients with Down syndrome, and the cause for this is unknown. The majority of patients present in their first decade, with the clinical presentation varying with age. The cause of moyamoya syndrome in people with trisomy 21 is unknown. This research aimed to learn more about the genesis and pathology of moyamoya syndrome in people with Down syndrome.

The Preferred Reporting Items for Systematic Review and Meta-Analysis (PRISMA) guidelines were used to conduct this systematic review. Several publications connected to this topic were searched through a comprehensive database search. They were narrowed down to a final number of ten articles after applying inclusion and exclusion criteria and analyzing the quality of each work. Several possibilities were presented in these final papers to explain the link between moyamoya syndrome and trisomy 21. Trisomy 21 patients have a genetic predisposition to vascular problems. The RNF213 gene may interact with the genes on chromosome 21 that influence vascular physiology and elasticity in patients with Down syndrome, resulting in the whole picture of moyamoya syndrome.

## Introduction and background

Down syndrome is a highly common chromosomal defect in approximately one out of every 800 live births [1]. Down syndrome is primarily caused by chromosome 21 trisomy. Individuals with Down syndrome now have a life expectancy of roughly 60 years [2]. For a variety of reasons, including vascular abnormalities and congenital heart disease, patients with Down syndrome are vulnerable to cerebrovascular accidents. Moyamoya disease is uncommon, progressive stenosis of many cerebral arteries [3]. As a result of this blockage, a vascular network grows around the stenosed vessel. Collaterals are small and delicate vessels that are prone to bleeding, aneurism, or thrombus formation. On standard angiographic imaging, this vascular network appears as a "puff of smoke" (referred to in Japanese as the moyamoya phenomenon) [3]. Moyamoya is classified into two subtypes: idiopathic moyamoya illness and moyamoya syndrome. The vascular alterations in moyamoya syndrome are frequently associated with other syndromes or systemic disorders, examples include Down syndrome, sickle cell anemia, neurofibromatosis type-1, congenital heart disease, fibromuscular dysplasia, activated protein C resistance, and head trauma [4-5]. Moyamoya syndrome in trisomy 21 might manifest clinically as transitory ischemia symptoms or as a neurological disability. Other people may present with no symptoms, and these distinctive vascular alterations may be discovered by chance. Typically, standard angiographic imaging is used to confirm the diagnosis. Moyamoya syndrome in trisomy 21 was poorly understood in terms of its origin and pathophysiology. This comprehensive review attempts to gain a better understanding of the disease's pathophysiology in Down's syndrome.

## Review

Methods

The findings were reported in line with the Preferred Reporting Items for Systematic Review and Meta-Analysis (PRISMA) criteria and principles, and the systematic review was conducted in accordance with these standards and principles [6].

Search Strategy

PubMed, PubMed Central (PMC), and Google Scholar were employed as major research literature databases and search engines. The search was conducted utilizing key terms and a medical subject heading (MeSH) method, which might have resulted in the discovery of numerous papers demonstrating a link between moyamoya syndroma and Down syndrome. "Down syndrome" and "Moya Moya syndrome" were the key phrases utilized in the literature search. The MeSH strategy used in PubMed and PMC for the previously listed key words was: ("Down syndrome/cerebrospinal fluid" [MeSH] OR "Down syndrome/complications" [MeSH] OR "Down syndrome/diagnosis" [MeSH] OR "Down syndrome/diagnostic imaging" [Mesh] OR "Down syndrome/etiology" [MeSH] OR "Down syndrome/genetics" [MeSH] OR "Down syndrome/pathology" [MeSH] OR "Down syndrome/physiology" [MeSH] OR "Down syndrome/physiopathology" [MeSH]) AND ("moyamoya disease" [MeSH terms] OR ("moyamoya" [All Fields] AND "disease" [All Fields]) OR "moyamoya disease" [All Fields] OR "moyamoya" [All Fields]) OR (idiopathic [All Fields] AND "progressive" [All Fields] AND occlusive [All Fields] AND ("disease" [MeSH terms] OR "disease" [All Fields]) AND ("circle of willis" [MeSH terms] OR ("circle" [All Fields] AND "willis" [All Fields]) OR "circle of willis" [All Fields])) OR ("moyamoya disease 1" [All Fields] OR "spontaneous occlusion of the circle of willis" [All Fields]) OR (("cerebrum" [MeSH terms] OR "cerebrum" [All Fields] OR "cerebral" [All Fields] OR "brain" [MeSH terms] OR "brain" [All Fields]) AND basal [All Fields] AND rete [All Fields] AND mirabile [All Fields]) OR (("cerebrum" [MeSH terms] OR "cerebrum" [All Fields] OR "cerebral" [All Fields] OR "brain" [MeSH terms] OR "brain" [All Fields]) AND ("telangiectasis" [MeSH terms] OR "telangiectasis" [All Fields] OR "telangiectasia" [All Fields])) OR ("moyamoya sisease/blood" [Majr] OR "moyamoya disease/cerebrospinal fluid" [Majr] OR "moyamoya disease/classification" [Majr] OR "moyamoya Disease/complications" [Majr] OR "moyamoya disease/diagnosis" [Majr] OR "moyamoya disease/diagnostic imaging" [Majr] OR "moyamoya disease/etiology" [Majr] OR "moyamoya disease/genetics" [Majr] OR "moyamoya disease/immunology" [Majr] OR "moyamoya disease/pathology" [Majr] OR "moyamoya disease/physiopathology" [Majr]).

After the first screening, the total number of papers found in PubMed/Medline was decreased to 393 from 4185. Additionally, a search of the PMC database revealed 12195 papers, which were filtered down to 259. On Google Scholar, approximately 11 papers related to this systematic review were discovered. Additional databases and search engines, such as Cochrane, Web of Science, EBSCO, Popline, and CHBD, did not yield any papers relevant to our inquiry. Grey literature was omitted from this analysis because of the critical need to conduct a validated study.

Eligibility Criteria

The following criteria were used to choose the literature papers that were included in this systematic review:

Inclusion criteria: Papers that have been published within the last 21 years; accepting only full-text papers; patients who exhibit characteristics that are consistent with Down syndrome; findings on imaging suggestive of moyamoya syndrome; symptoms of a stroke ranging from transitory ischemia episodes to permanent neurological impairments; patient with no signs or symptoms of a stroke but a diagnosis of moyamoya syndrome on imaging.

Exclusion criteria: Other causes of cerebrovascular events; papers published more than 21 years ago.

Article Screening and Asses for Eligibility

First, records were screened by title or abstract to weed out those that were deemed unqualified. After that, a set of inclusion and exclusion criteria was used to sort through the remaining data. The remaining articles were read in detail and were checked for quality. The articles that satisfied the quality check were finalized to be included in the review.

PRISMA Flow Diagram

Around 663 items were narrowed down after the initial screening of the identified papers. Approximately 169 duplicate items were eliminated. Then, publications were filtered by title or abstract, and some studies were removed due to a lack of full-text articles and/or irrelevant articles. After evaluating 50 publications for eligibility, only 10 were included in the study.

**Figure 1:**
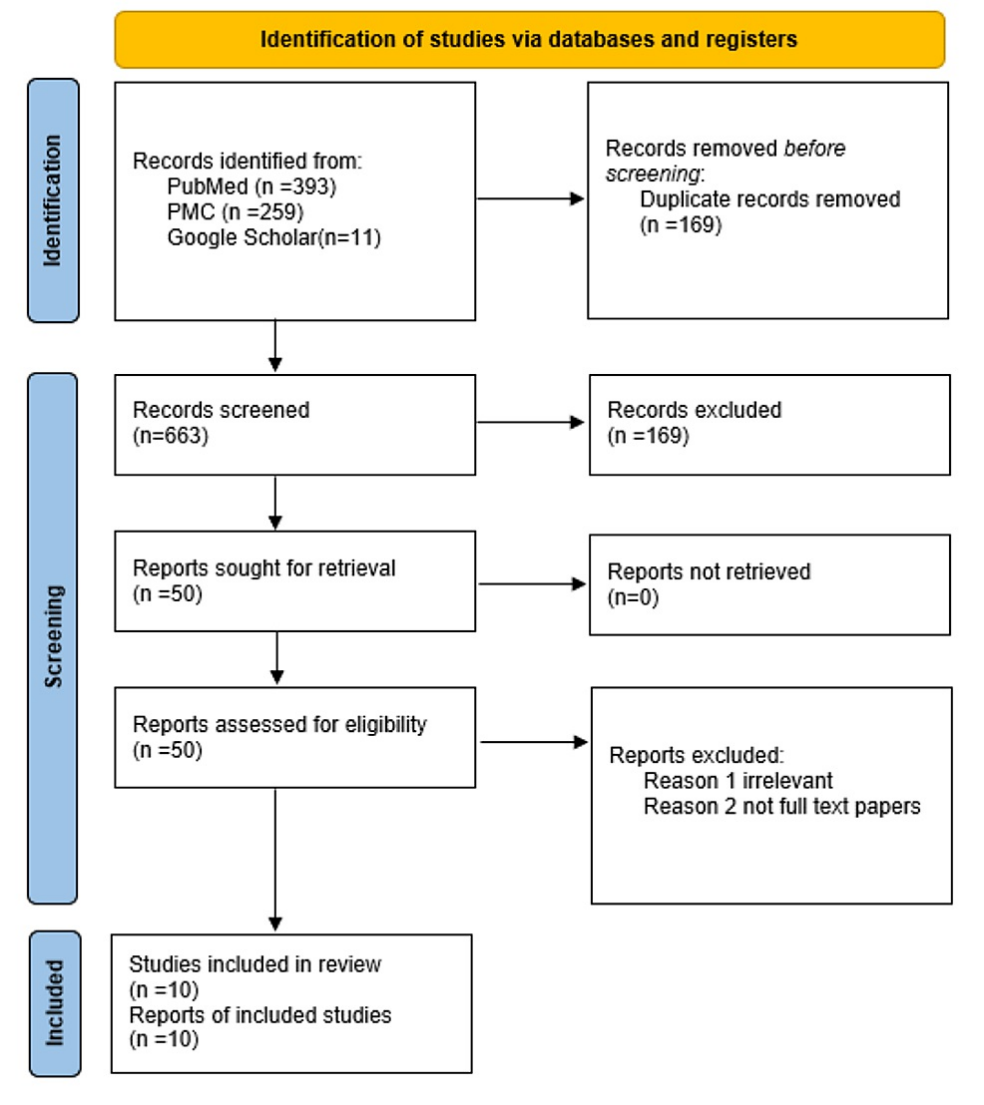
PRISMA 2020 flow diagram for systematic review PRISMA: Preferred Reporting Items for Systematic Review and Meta-Analysis; PMC: PubMed Central.

Figure 1 below depicts the flow diagram for article selection based on PRISMA.

Quality Appraisal of Studies

This systematic review comprised three types of studies: a case report, a case series, and a meta-analysis. The quality of the case reports was assessed using the Joanna Briggs Institute (JBI) check tool, and the quality of the case series was assessed using the National Institutes of Health (NIH) quality assessment tool. A quality assessment was performed using the assessment of multiple systematic reviews (AMSTAR) tool on the meta-analysis study part of this systematic review. This literature review included all articles with a score of 60% or above. The findings are summarized in the following tables (Tables 1-3).

**Table 1:** Joanna Briggs Institute (JBI) critical appraisal tool for case reports and case study (2017)

Study/year of publication	Were the patient's demographic characteristics clearly described?		Was the patient's history clearly described and presented as a timeline?	Was the current clinical condition of the patient on presentation clearly described?	Were diagnostic tests or assessment methods and the results clearly described?	Was the intervention (s) or treatment procedure (s) clearly described?	Was the post-intervention clinical condition clearly described?	Were adverse events (harms) or unanticipated events identified and described?	Does the case report provide takeaway lessons?	Overall appraisal
Tajmalzai A et al. 2021 [7]		Yes	Yes	Yes	Yes	Yes	Un clear	NA	Yes	Include
Tavares Bello C et al. 2017 [8]		Yes	Yes	Yes	Yes	Yes	Yes	NA	Yes	Include
Mishra A et al. 2012 [9]		Unclear	Yes	Yes	Yes	No	No	NA	Yes	Include
Rison RA et al. 2008 [10]		Yes	Yes	Yes	Yes	Yes	Yes	NA	Yes	Include
Lee KY et al. 2013 [11]		Yes	Yes	Yes	Yes	NA	Yes	NA	Yes	Include
Sameshima T et al. 2000 [12]		Yes	Yes	Yes	Yes	Yes	Yes	NA	Yes	Include
Makwana M et al. 2017 [13]		Yes	Yes	Yes	Yes	Yes	Yes	NA	Yes	Include
Paneerselvam SP et al. 2019 [14]		Yes	Yes	Yes	Yes	Yes	No	NA	Yes	Include

**Table 2:** National Institutes of Health (NIH) quality assessment tool for case series

		Kumar P et al. 2018 [15]
1-Was the research topic or goal stated clearly?		Yes
2-Did the study population, including a case definition, have a clear and complete description?		Yes
3-Were the case consequetive?		Yes
4-Did the topics have any parallels?		No
5-Was the intervention clearly described?		Yes
6-Did the outcome measures clearly defined, were they valid, reliable, and used consistently across all study participants?		Yes
7-Was the follow-up period long enough?		Yes
8- Had all statistical procedures been thoroughly described?		Yes
9-Did the results come across as well-written?		Yes
10-How would you rate your overall performance?		Include

**Table 3:** Assessment of multiple systematic reviews (AMSTAR) assessment tool for meta-analysis

Study	Junqueira PA et al. 2002 [16]
1-Was the study question clearly described in the publication, and were the inclusion and exclusion criteria listed?	Yes
2- Did you conduct a thorough review of the literature?	Yes
3-It should have been chosen by at least two people?	Yes
4-Was the publication's status used as a factor for inclusion?	No
5- Were there at least two participants who were not included in the study?	Can't say
6-Was the study that was excluded listed?	No
7-Did the relevant characteristics of the studies that were included in the analysis be provided?	Yes
8-Was the scientific quality of the research included in the review evaluated and reported?	Yes
9-Was the scientific quality of the studies included in the analysis adequately used?	Yes
10-Did the right Methods for combining the findings of each particular study?	Yes
11-Was the likelihood of publication bias properly assessed?	Yes
12-Was there a declaration of a conflict of interest?	Yes
13-Overall evaluation	Include
14-Do the findings of this study apply directly to the patient population targeted by this guideline?	Yes

Results

Two independent authors extracted data from the finalized studies for this systematic review. The previous studies identified a total of 54 cases and recorded the following clinical and epidemiological data (Tables 4-9).

**Table 4:** Gender

Gender	
23 males (42.59%)	31 females (57.40%)

**Table 5:** Age range of participants in this systematic review

Age of the patients included in this systematic review		
Grouping by age	The total number of patients	Percentage
Up to the age of 12	44	81.48%
13–19 years of age	3	5.55%
Over the age of 20	7	12.96%

**Table 6:** Clinical presentation

Clinical manifestations		
Symptoms	Symptoms reported by patients	Percentage
Hemiparesis	42	77.77%
Speech disorder	12	22.22%
Seizure	9	16.66%
Involuntary movement	3	5.55%
Facial Paralysis	6	11.11%
Headache	3	6.55%
Optic atrophy and cortical blindness	1	1.85%
Altered Sensorium	1	1.85%
Hemihypersethia	1	1.85%
Estropia, amblyopia, Cyclotorsion of eyes, Horizontal nystagmus	1	1.85%
Urinary Incontinence	1	1.85%

**Table 7:** Presentational subtype

Presentational subtype	Number of the patients	percentage
Ischemic Stroke	42	77.77%
Transient ischemic attacks	8	14.81%
Hemorrhagic Stroke	4	7.40%
Intracerebral Haematoma with Intraventricular Extension	1	1.85%
Recurrent ischemic episode	30	55.55%
Isolated Ischemic Episode	21	38.88%

**Table 8:** Involvement of the vascular system

Involvement of the vascular system	Number of the patients	percentage
Bilateral	41	75.92%
Unilateral	9	16.66%
Not identified	4	7.40%

**Table 9:** The type of cardiac anomaly discovered in the participants included in this systematic review

Evaluation of cardiac abnormalities		
the nature of the cardiac abnormality	Number of the patients	percentage
Congenital heart disease	12	22.22%
Normal heart	2	3.70%
Not identified	40	74.07%

The majority of the instances featured in this research were in females and occurred in the first decade. Ischemic stroke is more common in the first decade of life, although the clinical picture changes as people get older. Hemorrhagic stroke is the most common type of stroke in adults. Moyamoya phenomena were found in all the angiography instances that were described. Many possibilities have been put up as to why moyamoya syndrome is linked to trisomy 21, and these will be investigated in greater depth later in this study. Understanding the pathophysiology of moyamoya syndrome in Down syndrome patients will aid in the treatment and prevention of recurrence of symptoms.

Discussion

A condition called moyamoya disease can rarely result in gradual worsening stenosis of one's internal carotid artery or the anterior, middle, or posterior cerebral arteries. An arterial network is formed around the site of stenosis to compensate, and this phenomenon is called the moyamoya phenomenon in Japanese. The syndrome was first described in Japanese patients, and its frequency is still very high in Japan, affecting roughly five out of 100,000 people. Moyamoya syndrome is characterized by vascular abnormalities that occur in conjunction with other systemic diseases or syndromes, such as thyrotoxicosis, leptospirosis, tuberculosis, aplastic anemia, Fanconi anemia, sickle cell anemia, lupus anticoagulant or antiphospholipid, Down syndrome, Marfan syndrome, tuberous sclerosis, Turner syndrome, and coarctation of the aorta. A greater proportion of Asians are infected with moyamoya illness. Females are nearly twice as likely as males to suffer from the condition. Among those with Down syndrome, moyamoya illness is three times more common than in the general population [17-18]. It is not clear why this is the case. Most patients present in the first decade with a smaller percentage appearing in the fourth decade [19-20]. It is a bilateral disease; however, there has been an increase in cases where just one side is affected.

Etiology and Pathology of Moyamoya in Trisomy 13

Inflammation or atherosclerosis was not found in the stenosed arteries. Smooth muscle hypertrophy, luminal thrombosis, and tunica media thinning are all observed in these stenosed arteries. When blood flow is raised because of the obstruction, a compensatory network of vascular vessels forms composed of both pre-existing and newly formed vessels, this suggests that the increased blood flow has stressed the vessels. The observed modifications are fragmented elastic lamina, thinning media, and microaneurysms. The underlying mechanism of moyamoya disease in trisomy 21 has been the subject of several hypotheses. There has been some discussion of a genetic cause for this connection. There is an increase in the expression of certain growth factors, enzymes, and peptides in patients with moyamoya illness. Some of these growth factors are transforming growth factor-1, hepatocyte growth factor, vasculature endothelial growth factor, and hypoxia-inducing factor-1 [21].

When it comes to blood vessels, people with Down syndrome are genetically predisposed to developing a wide range of anomalies, from an irregular nail bed capillary structure to pulmonary artery hypertension to retinal vessel abnormalities to renal artery hypertension [22]. Many proteins on chromosome 21 have an impact on arterial physiology and flexibility. Several proteins are implicated in oxidative stress, including superoxide dismutase-1, interferon-gamma receptor, and cystathionine synthase [23]. The low rate of atheroma in Down syndrome patients may be caused by one or more of these proteins [24-26]. Collagen type VI was found in the lining of large arteries. Vascular injury may boost its expression in cerebral arteries [27]. The alpha-chain collagen is encoded on chromosome 21. As a result, aberrant overexpression of collagen type VI in some cases of moyamoya syndrome may result in malformed blood vessels [28].

The RNF213 protein gene is frequently mutated in patients with moyamoya. The development of blood vessels is aided by this protein, which is found throughout the body. There are two distinct protein 'classes,' and RNF213 is a member of both. To begin with, it is an E3 enzyme in nature. This protein can identify undesired or malfunctioning proteins, and the cell can then be rid of them. A motor protein is also present. Small molecular "engines" that transform chemical energy into movement are found in motor proteins. Only the human protein RNF213 performs both of these tasks. In biochemical studies of the mutation most commonly observed in moyamoya patients, the overall structure, ATPase motor, and E3 module of RNF213 were all confirmed to be intact. Therefore, the disease-causing mutation appeared to affect interactions with other companion proteins rather than destroying RNF213 itself [29]. Nevertheless, an interaction between RNF213 and genes on chromosome 21 may result in moyamoya syndrome in persons with Down syndrome.

Histopathological Findings

There is an uneven elastic lamina in the sick vessel, surrounded by areas of significant endothelial hyperplasia, fibrous intimal thickening, tunica media with atrophic zones replaced by collagen, and damaged and thickened elastic layer [30].

Clinical Presentation and Diagnosis

Moyamoya syndrome clinical manifestations in Down syndrome patients vary with age. Ischemic stroke or transient ischemic episodes are more common in children at a higher risk of hemorrhagic stroke as they age. Symptoms include headache, involuntary movement, difficulty speaking, weakness in the arms and/or legs, cognitive impairment, and seizure. To confirm the diagnosis, the following criteria must be met:

(a) Stenosis of the distal intracranial portion of the internal carotid artery, or the proximal anterior cerebral artery, middle, or posterior cerebral artery.

(b) An abnormal vascular network developing near the stenosis from the thalamoperforate and lenticulostriate arteries.

(c) Rule out other possible causes, such as trauma, meningitis, sickle cell disease, tumors, or radiation therapy.

Cerebral angiography confirms the diagnosis by showing the distinct collateral development along the stenotic artery.

Treatment Options of Moyamoya Disease

Management of moyamoya syndrome is controversial. Some clinicians support medical treatment only, while others prefer the long-lasting protecting outcome of surgery.

The treatment options are divided into medical and surgical. Medical management includes aspirin. At the same time, the surgical intervention involves several revascularization procedures. These are encephaloarteriosynangiosis, encephalomyosynangiosis, superficial temporal artery to middle cerebral artery anastomosis, and omental transplantation. Cerebral revascularization surgery with the pial synangiosis technique provides a better outcome with reduced future risk of stroke compared to other techniques [31-34]. There is no rule for anticoagulant medication in secondary prevention as the risk of bleeding in these patients is high.

Limitations

The limitation of this systematic review was the limited number of studies examining the underlying mechanism of moyamoya syndrome. Additionally, the majority of the papers discovered were case reports. In addition, some patients were followed for a short period of time, while others were difficult to track down for an extended period of time.

## Conclusions

The association of moyamoya syndrome with trisomy 21 has been increasingly recognized over the last few years. The reason for this association is yet to be understood, with many hypotheses, most of them pointing toward genetic predisposition linking moyamoya syndrome and trisomy 21. The RNF213 gene may interact with the genes on chromosome 21 that influence vascular physiology and elasticity in patients with Down syndrome, resulting in the picture of moyamoya syndrome. Further research is needed to confirm or rule out this association. In patients with Down syndrome who present with neurological deficits, a diagnosis of moyamoya syndrome should always be kept in mind, and the diagnosis should be confirmed with cerebral angiographic studies.
